# Cluster analysis of angiotensin biomarkers to identify antihypertensive drug treatment in population studies

**DOI:** 10.1186/s12874-023-01930-8

**Published:** 2023-05-27

**Authors:** Maeregu Woldeyes Arisido, Luisa Foco, Robin Shoemaker, Roberto Melotti, Christian Delles, Martin Gögele, Stefano Barolo, Stephanie Baron, Michel Azizi, Anna F. Dominiczak, Maria-Christina Zennaro, Peter P. Pramstaller, Marko Poglitsch, Cristian Pattaro

**Affiliations:** 1 Institute for Biomedicine (affiliated to the University of Lübeck), Eurac Research, Via Volta 21, 39100 Bolzano, Italy; 2 Department of Dietetics and Human Nutrition, University of Kentucky, Lexington, USA; 3 School of Cardiovascular and Metabolic Health , University of Glasgow, Glasgow, UK; 4 Hospital of Schlanders/Silandro, Schlanders/Silandro, Italy; 5 National Institute of Health and Medical Research (Inserm), Paris, France; 6 Hypertension Department and DMU CARTE, AP-HP, Hôpital Européen Georges-Pompidou, Paris, France; 7 Attoquant Diagnostics, Vienna, Austria; 8 Health Data Science Center, Human Technopole, Viale Rita Levi Montalcini, 1, 20157 Milan, Italy; 9 Université Paris Cité, Paris, France

**Keywords:** Angiotensin, Aldosterone, Antihypertensive drugs, Cluster analysis, Lasso regression, CHRIS study

## Abstract

**Background:**

The recent progress in molecular biology generates an increasing interest in investigating molecular biomarkers as markers of response to treatments. The present work is motivated by a study, where the objective was to explore the potential of the molecular biomarkers of renin-angiotensin-aldosterone system (RAAS) to identify the undertaken antihypertensive treatments in the general population. Population-based studies offer an opportunity to assess the effectiveness of treatments in real-world scenarios. However, lack of quality documentation, especially when electronic health record linkage is unavailable, leads to inaccurate reporting and classification bias.

**Method:**

We present a machine learning clustering technique to determine the potential of measured RAAS biomarkers for the identification of undertaken treatments in the general population. The biomarkers were simultaneously determined through a novel mass-spectrometry analysis in 800 participants of the Cooperative Health Research In South Tyrol (CHRIS) study with documented antihypertensive treatments. We assessed the agreement, sensitivity and specificity of the resulting clusters against known treatment types. Through the lasso penalized regression, we identified clinical characteristics associated with the biomarkers, accounting for the effects of cluster and treatment classifications.

**Results:**

We identified three well-separated clusters: cluster 1 (n = 444) preferentially including individuals not receiving RAAS-targeting drugs; cluster 2 (n = 235) identifying angiotensin type 1 receptor blockers (ARB) users (weighted kappa κ_w_ = 74%; sensitivity = 73%; specificity = 83%); and cluster 3 (n = 121) well discriminating angiotensin-converting enzyme inhibitors (ACEi) users (κ_w_ = 81%; sensitivity = 55%; specificity = 90%). Individuals in clusters 2 and 3 had higher frequency of diabetes as well as higher fasting glucose and BMI levels. Age, sex and kidney function were strong predictors of the RAAS biomarkers independently of the cluster structure.

**Conclusions:**

Unsupervised clustering of angiotensin-based biomarkers is a viable technique to identify individuals on specific antihypertensive treatments, pointing to a potential application of the biomarkers as useful clinical diagnostic tools even outside of a controlled clinical setting.

**Supplementary Information:**

The online version contains supplementary material available at 10.1186/s12874-023-01930-8.

## Background

The recent progress in molecular biology generates an increasing interest in investigating molecular biomarkers as markers of diagnosis, prognosis or response to treatment. For example, the present work is motivated by the molecular epidemiology of hypertension study, where the objective was to explore the potential of the molecular biomarkers of RAAS to identify the undertaken antihypertensive drug (AHD) treatments in the general population. Hypertension is a leading cause of death worldwide [1] and a primary risk factor for various comorbidities [2]. RAAS targeting AHD treatments are central to the treatment of hypertension, which include angiotensin-converting enzyme inhibitors (ACEi) and angiotensin receptor blockers (ARB), either monotherapy or combined with a diuretic. Combinations of an ACEi or an ARB with a calcium channel blocker or a diuretic is the recommended first-line treatment for hypertension [3].

Population-based epidemiological studies provide an opportunity to assess AHD effectiveness in real non-clinical contexts. While these studies generally have a much larger scale than clinical controlled studies, they often lack high-quality documentation of the AHD treatment, especially when linkage to electronic health records is not available. In the absence of efficient drug information retrieval systems, treatment self-reporting is imprecise and subject to classification bias [4, 5]. On the other hand, sample biobanking guarantees the possibility to measure extensive sets of molecular biomarkers afterwards and, for example, to reconstruct the most likely AHD treatment *a posteriori* using specific statistical methods. This was possible thanks to the recent advance in modern techniques such as liquid chromatography combined with tandem mass spectrometry (LC-MS/MS) to simultaneously measure the RAAS biomarkers in biobanked blood samples [6].

In the present study, we investigated whether unsupervised cluster analysis of biomarkers of the RAAS may help to identify the undertaken AHD treatment. To this end, we measured the three RAAS biomarkers angiotensin I, angiotensin II and aldosterone using LC-MS/MS [6] in biobanked serum samples from 800 participants from the Cooperative Health Research In South Tyrol (CHRIS) study [7], where AHD classification was constructed accurately through automatic drug package barcode scanning upon participation.

We evaluated the agreement between estimated clusters and the objective classification obtained via drug box barcode scanning. The resulting clusters were characterized based on available clinical information. Finally, to identify possible reasons of imperfect classification, we performed a lasso penalized regression and assessed which clinical characteristics, among those typically associated with different AHD treatments, were related to each biomarker while accounting for the effects of the clustering and the AHD treatments.

## Methods

### Study design and participants

This study was based on the CHRIS study, a single-center, population-based study designed to investigate the molecular, behavioral and environmental determinants of human health, whose baseline assessment was carried out between 2011 and 2018 [7, 8]. Blood was collected from CHRIS study participants following overnight fasting. After immediate pre-analytical sample processing, sample storage at − 80 °C was performed as described in [7, 8]. Health-related information was collected through either self- or interviewer-administered interviews based on standardized electronic questionnaires. Participants were requested to bring any boxes of medications taken in the preceding week to the study center. Drug information was retrieved via scanning the drug box barcodes, automatically classified according to the Farmadati database (https://www.farmadati.it/), and stored in the CHRIS database.

At the time when the present study was set up, the CHRIS study included N = 6075 participants. Budget limitations allowed to measure the RAAS biomarkers on a random sample of 800 samples. Taking into account that the smallest treatment group included 100 individuals, we sampled 8 age- and sex-matched groups based on the AHD treatment: (1) normotensive; (2) untreated hypertensive; (3) participants taking other drugs not prescribed as AHD (referred to as *non-AHD*); (4) participants on ACEi monotherapy; (5) participants on ACEi combined with diuretics (*ACEi + diuretics*); (6) participants on ARB monotherapy; (7) participants on ARB in combination with diuretics (*ARB + diuretics*); and (8) participants on beta blocker monotherapy treatment (*Beta blockers*). The diuretic used in the single-pill combinations was always hydrochlorothiazide. Additionally, 5 participants in *ACEi + diuretics* and 5 in the *ARB + diuretics* were taking furosemide.

### Clinical characteristics

Blood pressure (BP) was measured in supine position with the Omron digital automatic BP Monitor M10-IT at the end of a 20-minute resting electrocardiogram. The mean of three measurements taken at 2 minutes intervals was recorded. Hypertension was defined as: reporting the use of an AHD (ATC codes starting with C02, C03, C04, C07, C08, and C09) or having a diastolic BP (DBP) of ≥ 90 mmHg or a systolic BP (SBP) of ≥ 140 mmHg, according to established guidelines [3]. Diabetes mellitus (DM) was defined as a positive answer to the question “*Do you have diabetes mellitus?*” or the reporting of glucose-lowering drugs (ATC codes: A10) or by measured levels of glycated haemoglobin (HbA1c) ≥ 6.5% (7.8 mmol/L) or glucose ≥ 126 mg/dl (7 mmol/L) [9]. Estimated glomerular filtration rate (eGFR) was obtained from serum creatinine using the CKD-EPI formula [10]. Serum levels of total cholesterol, cortisol, potassium and sodium were determined as previously described [8].

### Quantification of RAAS biomarkers

Equilibrium Angiotensin I, angiotensin II and aldosterone levels were simultaneously determined using RAAS Triple A testing (Attoquant Diagnostics GmbH, Vienna, Austria) via liquid chromatography combined with tandem mass spectrometry (LC-MS/MS) analysis as previously described [6]. Briefly, equilibration of serum samples was performed at 37 °C for one hour, followed by stabilization through addition of an enzyme inhibitor cocktail. Samples were spiked with stable isotope-labeled internal standards for each analyte, and subjected to C-18-based solid-phase-extraction followed by LC-MS/MS analysis using a reversed-phase analytical column operating in line with a Xevo TQ-S triple quadruple mass spectrometer (Waters). Internal standards were used to correct for peptide recovery of the sample preparation procedure for each analyte in each individual sample.

The biomarkers were quantified from integrated chromatograms considering the corresponding response factors determined in appropriate calibration curves in serum matrix, on condition that integrated signals exceeded a signal-to-noise ratio of 10. The lower limits of quantification for angiotensin I, angiotensin II and aldosterone, defined as the lowest concentrations tested showing a coefficient of variation (CV) < 20% according to FDA criteria, are 5 pg/ml for each of the three biomarkers, corresponding to 3.9, 4.8 and 13.9 pmol/L, respectively. At 50 pmol/L, the inter-assay CVs for the three biomarkers are 10.2%, 6.1%, and 7.9%, respectively, while the corresponding intra-assay CVs are 8.6%, 4.4%, and 5.2%, respectively.

### Statistical analyses

**Fig. 1:**
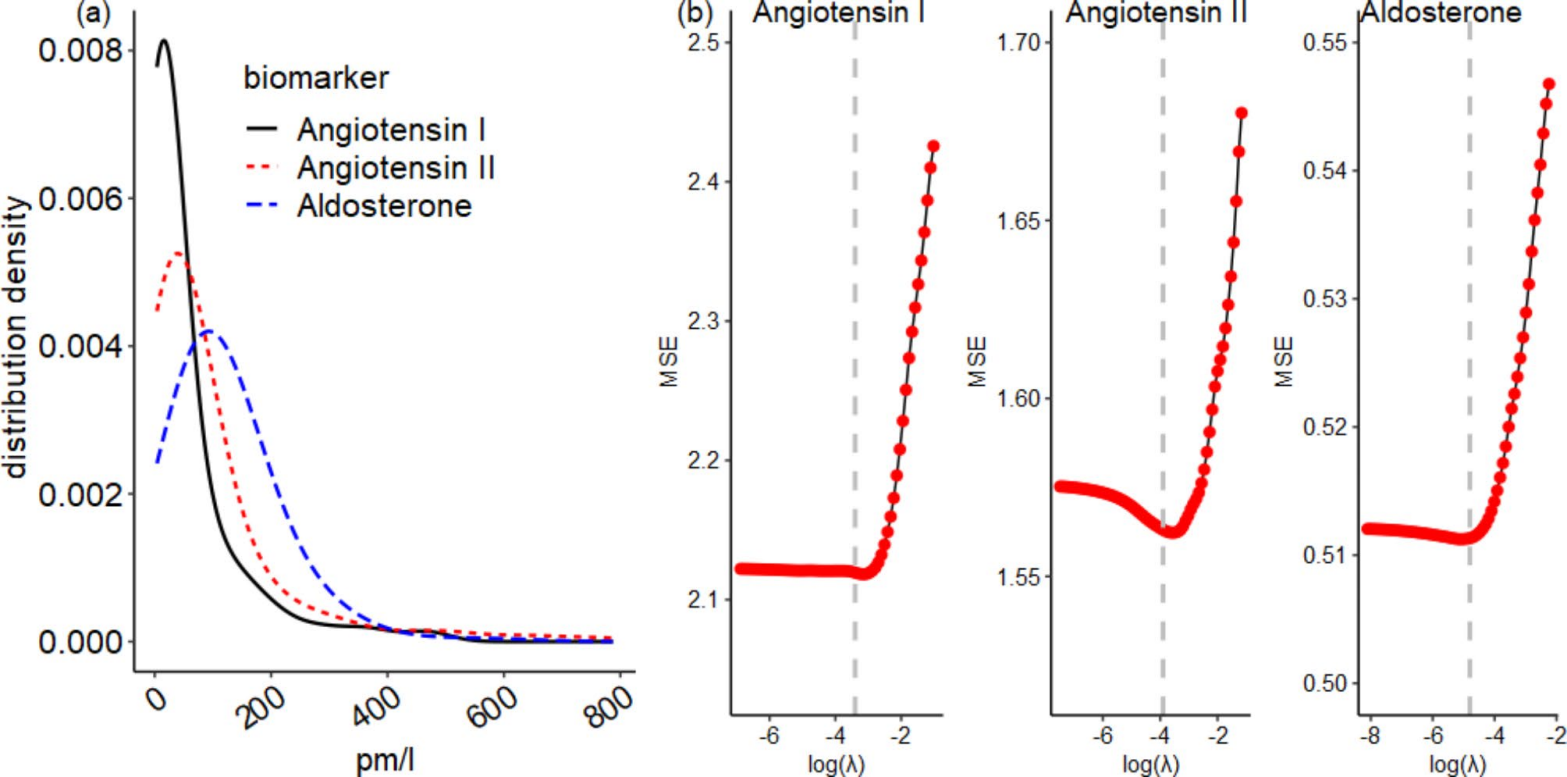
(a): Skewed non-normal distributions of Angiotensin I, Angiotensin II and Aldosterone in the study sample. The density lines were obtained using the Gaussian smoothing kernel. (b): Identification of the optimal penalty parameter λ (in log scale) for the lasso regression. Results are shown from the 8-fold cross-validation (CV). The vertical gray dashed lines represent the penalty parameter that achieved the least MSE.

The distributions of angiotensin I, angiotensin II and aldosterone were skewed to the left, hence they were log-transformed to achieve normality (Fig. 1a). First, to determine whether the three RAAS biomarkers could identify participants in different AHD groups, we conducted K-means unsupervised cluster analysis [11, 12] by assigning each observation to one of k groups based on a similarity feature computed from the biomarkers’ covariance matrix. Cluster membership is computed as the sum of the squared distance between data points and the cluster center using the Euclidean distance [13, 14]. We inspected the identified clusters using principal components (PCs), which were obtained as the linear combinations of the normalized three RAAS biomarkers and their corresponding loadings or weights. The k-means method was chosen after comparison with the alternative unsupervised machine learning approaches such as hierarchical and fuzzy clustering [15]. Selection of the best method as well as the optimal number of clusters was based on the *Silhouette* score [16], which was evaluated for a number of clusters between 2 and 6. We included a sensitivity analysis to determine whether the obtained optimal number of clusters remain the same if the candidate clusters were increased between 2 and 8.

We assessed the agreement between AHD treatment identified by the clusters and the eight groups using the weighted kappa (κ_w_) inter-agreement coefficient [17]. κ_w_ is a modification of the Cohen’s index [18] to deal with chance agreement between classifiers, and defined based on conditional probability that two classifiers will agree given that disagreement will occur by chance. Computational details are provided in [17]. Sensitivity and specificity were estimated considering the objective AHD classification obtained by the drug box barcode scanning as the gold standard.

Next, we assessed differences of the clinical characteristics among the clusters using one-way analysis of variance (ANOVA) or chi‐squared test where appropriate. If the ANOVA test indicated evidence of significant difference between clusters, we performed pairwise comparisons using Tukey multiple test correction procedure. Finally, we fitted a lasso penalized regression model [19] to assess whether any clinical predictor could explain the residual variance of each RAAS biomarker, which was not explained by the clusters or the treatment. We fitted a model for each biomarker as the response variable, setting clinical characteristics as fixed-effect predictors and the identified clusters and the treatment group as random-effect terms. The rationale to introducing the identified clusters and treatment groups as random effect was to capture the residual variability not accounted for by the fixed effect predictors.

The lasso penalization was applied to obtain a parsimonious predictive model that should not suffer from the between-predictor pairwise correlation (Supplementary Fig. 1): coefficients are constrained by imposing a penalty to drop the less influential predictors from the model by shrinking their coefficients to zero [19, 20]. The penalty level was tuned by selecting a penalty parameter $\lambda$ using k-fold cross-validation (CV), with the aim to minimize the mean squared error (MSE). We set k = 8 and the smallest MSE was observed at $\lambda$=0.03, 0.02 and 0.01, for angiotensin I, angiotensin II and aldosterone, respectively (Fig. 1b). The statistical significance level was set at 0.05 in all analyses. All analyses were performed with the R software v4.0.5, using the packages *stats* v4.2.0 and *cluster* v2.1.2 [21] for cluster analysis and *glmnet* v4.1.3 and *glmmLasso* v1.5.1 [22] for penalized regression analysis.

## Results

### Characteristics of study participants

Clinical characteristics of each group are described in Table 1. Study participants were 43 to 90 years old, and 54% were females. Normotensive participants had the lowest SBP and DBP levels, while the hypertensive group had the highest BP levels. In the treatment groups, mean BP was within the control limits, with exception of ACEi users, whose mean SBP levels were > 140 mmHg. AHD treatment groups showed larger BMI levels compared to normotensive individuals, with maximal BMI levels in the ARB + diuretics group. Additional clinical characteristics are reported in Supplementary Table 1.

**Table 1:** Characteristics of the 800 participants by AHD treatment group

AHD group	N	Sex M/F	Age, years mean(SD)	SBP, mmHg mean(SD)	DBP, mmHg mean(SD)	BMI, kg/m^2^ mean(SD)
Normotensive	101	46/55	64.9(7.2)	122.3(9.4)	78.7(6.4)	25.9(3.4)
Hypertensive	100	46/54	62.1(7.8)	146.7(12.2)	93.0(7.2)	27.2(4.4)
Non-AHD	100	46/54	68.8(10.1)	131.2(18.4)	81.0(8.3)	26.3(4.3)
Beta blockers	100	46/54	66.2(9.0)	134.6(16.8)	83.7(8.9)	28.2(4.8)
ACEi	99	45/54	68.7(10.0)	142.5(18.0)	85.5(9.4)	28.7(4.2)
ACEi + diuretics	100	47/53	68.6(10.0)	138.1(18.7)	84.0(8.2)	29.8(4.3)
ARB	98	45/53	65.2(9.5)	134.9(15.0)	84.7(8.4)	28.6(4.8)
ARB + diuretics	102	47/55	68.7(9.9)	139.9(17.9)	86.1(9.3)	30.5(5.6)
Overall	800	368/432	67(9.5)	136.3(17.5)	84.6(9.2)	28.1(4.7)

**Fig. 2:**
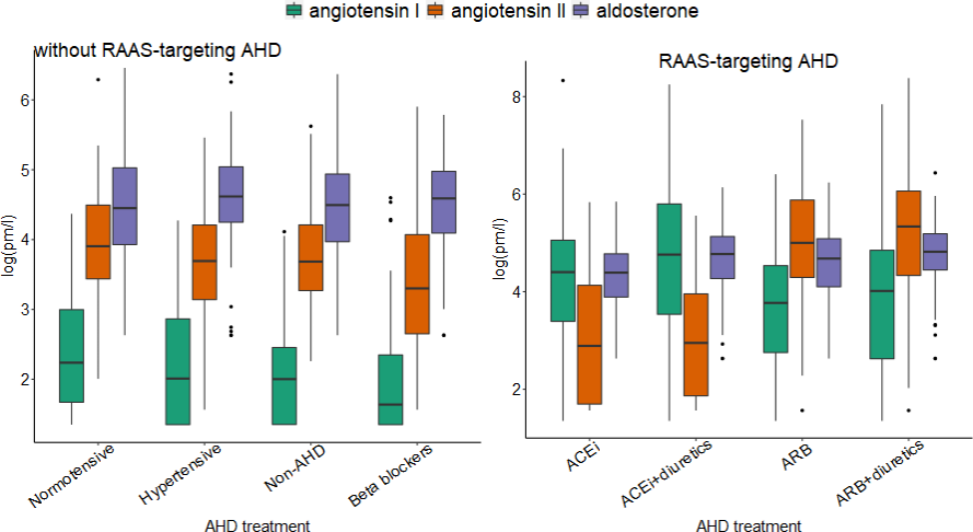
Distribution of angiotensin I, angiotensin II, and aldosterone according to AHD treatment status

Figure 2 illustrates the distributions of the three RAAS biomarkers across AHD groups. Angiotensin I, angiotensin II and aldosterone showed a similar joint profile in all AHD groups that do not include an ACEi or ARB (normotensive, hypertensive, non-AHD, and Beta blockers). In contrast, more elevated angiotensin I and depleted aldosterone levels were observed in the ACEi, ACEi + diuretics, ARB, and ARB + diuretics groups. Angiotensin II was depleted in the ACEi groups, and elevated in the ARB groups.

### Cluster analysis

**Fig. 3:**
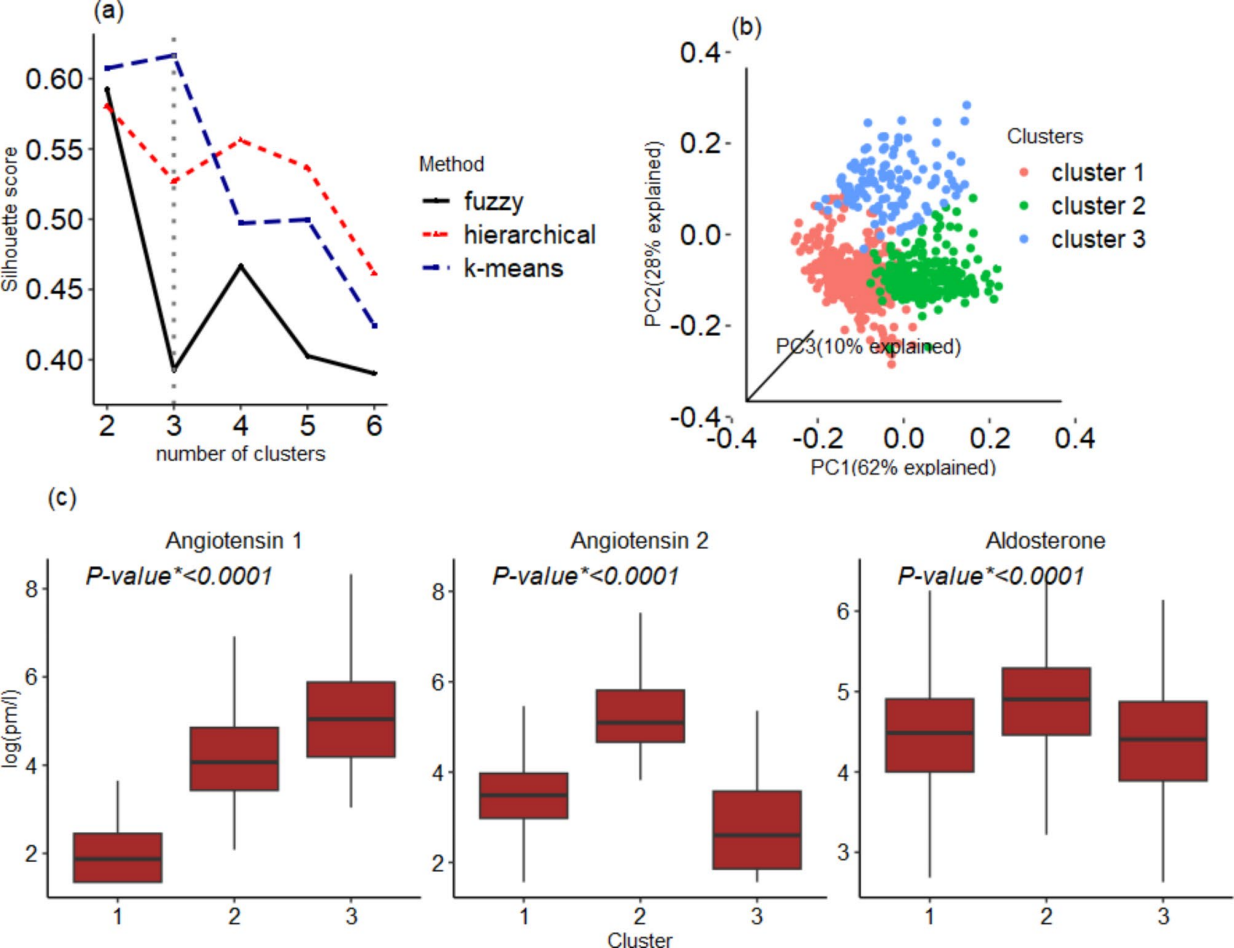
The unsupervised cluster analysis result. **Panel a**: identification of the optimal clustering solution via the Silhouette score metric. The dotted vertical line indicates that the k-means with three clusters provided the smallest (optimal) Silhouette score. **Panel b**: The resulting well-separated clusters; **Panel c**: Distribution of the three RAAS biomarkers across clusters. P-value* indicates P value computed from one-way ANOVA test

The Silhouette index analysis identified the unsupervised k-means clustering with three clusters as the optimal clustering solution compared to the alternative hierarchical and fuzzy clustering methods (Fig. 3a). The obtained optimal clustering solution remains the same for increased number of candidate clusters (supplementary Fig. 2). Consequently, the k-means cluster analysis of angiotensin I, angiotensin II, and aldosterone identified three well-separated clusters (Fig. 3b). The three PCs explained, respectively, 62%, 28%, and 10% of the RAAS biomarkers total variability. Cluster 1, 2, and 3, included 55%, 30% and 15% of the study participants, respectively. The three RAAS biomarkers showed substantially different distributions (one-way ANOVA test P < 0.0001) and distinct patterns over the three clusters (Fig. 3c): angiotensin I was lowest in cluster 1, intermediate in cluster 2 and largest in cluster 3, with non-overlapping distributions between clusters 1 and 3. Angiotensin II peaked in cluster 2 and showed lowest levels in cluster 3, with nearly non-overlapping distribution between clusters 2 and 3. Aldosterone was relatively less variable across the clusters, yet the difference between the clusters was statistically significant.

### Identification of AHD group by clusters

**Fig. 4:**
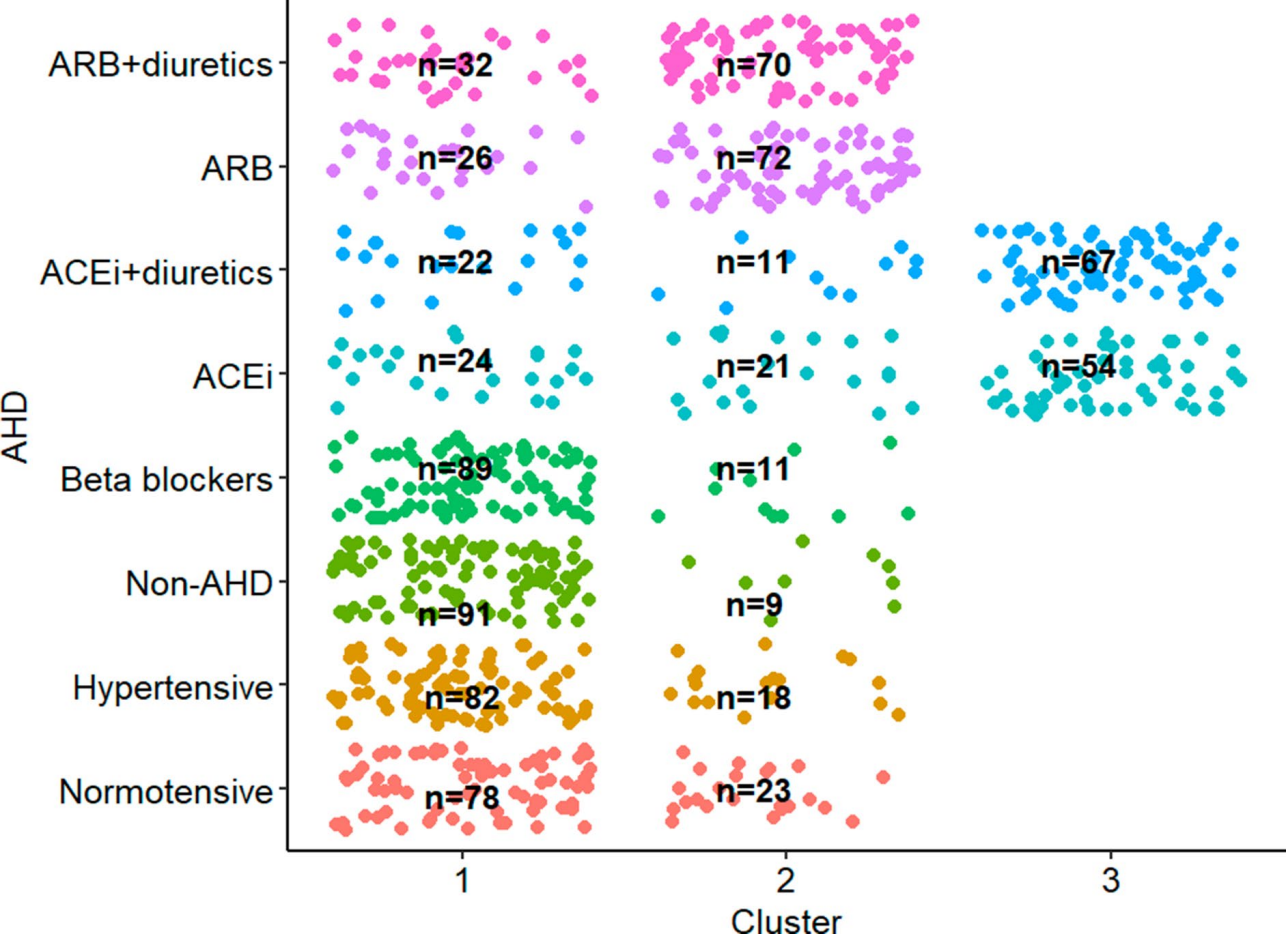
Distribution of study participants across eight AHD treatment status groups against the three identified clusters. The numbers represent the number of participants stratified by clusters by AHD groups

We evaluated to what extent the three clusters were able to identify different AHD treatment groups. Figure 4 depicts the study participants stratified by the eight AHD groups and by clusters. Cluster 1 comprised individuals from all groups but with strong preponderance of individuals from the normotensive, hypertensive, non-AHD, and beta blockers groups, that is, cluster 1 seems to represent individuals who are not on RAAS-targeting treatment (ATC = C09). Cluster 2 was enriched for individuals on ARB with or without diuretics. Cluster 3 included only individuals on ACEi with or without diuretics.

Results of agreement, sensitivity and specificity along with 95% confidence intervals (CI) from the analysis of the classification properties of the three clusters are shown in Table 2. We observed highest agreement between cluster 3 and ACEi (κ_w_ = 82%), with 55% sensitivity and 90% specificity, and between cluster 3 and ACEi + diuretics (κ_w_ = 78%, sensitivity = 67%, specificity = 92%). When joining ACEi and ACEi + diuretics groups together, cluster 3 showed κ_w_ = 82% (95%CI: 79-86%), 61% (95%CI: 54-68%) sensitivity, and 100% (95%CI: 99-100%) specificity. Cluster 2 had best agreement with ARB (κ_w_ = 74%, sensitivity = 73%, specificity = 83%) and ARB + diuretics (κ_w_ = 51%, sensitivity = 69%, specificity = 76%). When joining ARB and ARB + diuretics groups together, cluster 2 showed 71% (95%CI: 64-77%) sensitivity and 84% (95%CI: 81-87%) specificity. Cluster 1 showed a sensitivity between 77% and 91% to identify individuals from the normotensive, hypertensive, non-AHD and beta blocker groups (sensitivity = 85% when joining the four groups together). Specificity of cluster 1 to assess that an individual is either normotensive, hypertensive, non-AHD or beta blocker user but not a RAAS AHD user was 74% (95%CI: 69-78%).

**Table 2:** Weighted kappa, sensitivity and specificity along with the 95% CI for identification of AHD treatment between RAAS-generated clusters and AHD classification via scanning drug boxes

	Weighted kappa			Sensitivity			Specificity		
AHD group	Cluster1	Cluster2	Cluster3	Cluster1	Cluster2	Cluster3	Cluster1	Cluster2	Cluster3
Normotensive	63(58–68)	46(39–52)	12(4–19)	77(68–85)	23(15–32)	0(0–4)	48(44–51)	70(66–73)	83(80–85)
Hypertensive	64(59–69)	44(37–50)	14(6–21)	82(73–89)	18(11–27)	0(0–4)	48(45–52)	69(65–72)	83(80–85)
Non-AHD	60(56–66)	41(34–48)	18(10–25)	91(84–96)	9(4–16)	0(0–4)	50(46–53)	68(64–71)	83(80–85)
Beta blockers	45(39–52)	22(17–28)	6(1–12)	89(81–94)	11(6–19)	0(0–4)	49(46–53)	49(46–53)	0(0–4)
ACEi	12(4–20)	45(39–52)	82(78–85)	24(16–34)	21(14–31)	55(44–65)	40(36–44)	69(66–73)	90(88–93)
ACEi + diuretics	25(19–31)	22(17–28)	78(75–82)	22(14–31)	11(6–19)	67(57–76)	40(36–43)	68(64–71)	92(90–94)
ARB	23(18–29)	74(69–80)	45(39–49)	27(18–36)	73(64–82)	0(0–4)	40(37–44)	83(81–85)	83(80–85)
ARB + diuretics	21(15–27)	51(46–55)	44(38–51)	31(23–41)	69(59–77)	0(0–4)	41(37–45)	76(73–79)	83(80–85)
Non-RAAS*	23(16–31)	60(53–65)	16(8–24)	85(81–88)	15(12–19)	0(0–1)	74(69–78)	56(51–61)	70(65–74)
ACEi or ACEi + diur**	12(4–22)	45(38–54)	82(79–86)	23(17–30)	16(11–22)	61(54–68)	34(30–38)	66(62–70)	100(99–100)
ARB or ARB + diur***	70(64–74)	41(34–48)	27(19–34)	29(23–36)	71(64–77)	0(0–2)	36(32–40)	84(81–87)	80(76–83)

* participants not on RAAS targeting drugs

**group of participants who received the RAAS targeting ACEi or ACEi + diuretics

***Participants who received the RAAS targeting ARB or ARB + diuretics

### Clinical features of the clusters and association with RAAS biomarkers

The clinical characteristics of each cluster are described in Table 3. Cluster 1 was characterized by higher average SBP and DBP than clusters 2 and 3. Individuals in clusters 2 and 3 displayed more cardiometabolic abnormalities. Cluster 2 participants had elevated fasting blood glucose and HbA1c levels. Consistently, this cluster had the largest proportion of individuals with DM. Cluster 3 participants had lower eGFR and higher BMI than the other clusters. Clusters didn’t differ in terms of cholesterol, cortisol, sodium and potassium levels. For BMI, HbA1c, Glucose, eGFR, SBP, DBP showing evidence of difference between clusters from the one-way ANOVA, post-hoc pairwise comparisons were performed adjusting for multiple testing. BMI is higher in participants taking ARB and ACEi enriched groups, compared with cluster 1, and eGFR is lower in participants taking ARB and ACEi enriched groups, compared with cluster 1. SBP is lower in ARB compared with cluster 1. The results were reported in supplementary Table 2.

**Table 3:** Distribution of clinical and laboratory characteristics across the three clusters. Continuous variables are presented as mean (sd); categorical variables are presented as counts and percentages (%)

Variable	Cluster 1 (n = 444)	Cluster 2 (n = 235)	Cluster 3 (n = 121)	P-value*
Age (Year)	66.57(9.1)	65.71(9.7)	68.6(10.5)	0.1826
Sex F: n(%)	240(54.0)	129(55.0)	63(52.0)	0.8789
BMI (kg/m^2^)	27.4(4.5)	29.0(4.9)	29.3(4.5)	0.0021
HbA1c (%)	5.91(0.47)	6.04(0.63)	6.04(0.52)	1.05 × 10^− 6^
Glucose (mg/dl)	98.76(15)	104.38(23.12)	102.48(15.69)	4.42 × 10^− 5^
Total cholesterol (mg/dl)	224.19(41.68)	220.01(44.99)	221.36(41.83)	0.3235
eGFR (ml/min/1.73m^2^)	78.34(12.47)	74.51(14.85)	73.55(15.77)	0.0070
Sodium (mmol/L)	141.1(2.19)	140.73(2.49)	140.53(2.23)	0.8505
Potassium (mmol/L)	4.59(0.38)	4.58(0.41)	4.59(0.42)	0.7828
Cortisol (µg/dl)	13.53(4.52)	13.69(4.73)	13.59(4.89)	0.0967
SBP (mmHg)	137.99(18.46)	132.4(14.63)	137.54(17.9)	0.0434
DBP (mmHg)	85.27(9.4)	83.56(8.5)	83.96(9.42)	0.0019
Diabetes: n(%)	42(9%)	51(22%)	25(21%)	1.45 × 10^− 5^

*From one-way ANOVA for continuous and Chi-square test for categorical variables

If, after removing both the cluster and the treatment effects, the clinical characteristics considered above were still associated with angiotensin I, angiotensin II and aldosterone levels, that would indicate the presence of variability not captured by the two classifiers (clusters, treatment) and thus possible reasons for imperfect agreement between them. The lasso model fitting results are shown in Table 4. After removing the effect of the clusters and the treatment, angiotensin I was still associated with age, sex, eGFR, DBP, and DM. Angiotensin II was still associated with age, sex, eGFR, and SBP. Aldosterone was associated with sex, BMI, eGFR, and cortisol.

**Table 4:** Clinical predictors of angiotensin I, angiotensin II and aldosterone after accounting for cluster structure and AHD treatment

Variables	Angiotensin I		Angiotensin II		Aldosterone	
	Est(SE)	P-value	Est(SE)	P-value	Est(SE)	P-value
Age, year	-0.025(0.005)	2.17 × 10^− 6^	-0.017(0.005)	0.0012	removed*	
Sex M	0.416(0.087)	2.18 × 10^− 6^	0.308(0.077)	7.85 × 10^− 5^	-0.222(0.054)	4.95 × 10^− 5^
BMI kg/m^2^	0.018(0.01)	0.0666	0.014(0.009)	0.1353	0.016(0.006)	0.0113
HbA1c %	-0.109(0.101)	0.2839	removed*		removed*	
Glucose mg/dl	removed*		0.001(0.003)	0.8870	0.003(0.002)	0.1492
Total cholesterol, mg/dl	0.002(0.001)	0.0743	removed*		removed*	
eGFR ml/min/1.73m^2^	-0.013(0.004)	0.0002	-0.007(0.003)	0.0399	-0.006(0.002)	0.0013
Sodium, mmol/L	-0.034(0.018)	0.0627	-0.026(0.016)	0.1095	0.002(0.012)	0.8324
Potassium, mmol/L	0.135(0.113)	0.2356	0.119(0.102)	0.2417	-0.055(0.072)	0.4416
Cortisol, µg/dl	removed*		0.008(0.008)	0.3281	0.012(0.006)	0.0443
SBP, mmHg	removed*		-0.015(0.004)	2.69 × 10^− 5^	removed*	
DBP, mmHg	-0.029(0.005)	3.70 × 10^− 8^	-0.007(0.007)	0.2694	0.001(0.003)	0.7244
DM	0.303(0.154)	0.0493	0.178(0.13)	0.1719	-0.074(0.092)	0.4215

Est: estimate of the coefficient of association; SE: standard error of estimated coefficient

*removed by the lasso penalization algorithm when a predictor is less influential

## Discussion

We investigated to what extent unsupervised cluster analysis applied to measured RAAS biomarkers may help identify individuals from the general population according to the most likely AHD treatment. Our results show that unsupervised clustering can reliably identify individuals on ACEi monotherapy or in combination with diuretics. To a lower extent, clustering can also identify ARB users, with or without diuretics. This is in line with several studies reporting changes in the biomarkers in response to RAAS targeting treatment [6, 23]. Furthermore, normotensive, untreated hypertensive and beta blockers groups were classified in the same cluster 1. This implies that the clustering based on the three biomarkers was able to separate those classified as agents acting on RAAS (ACEi and ARB), from the remaining groups, including beta blockers. Despite beta1-adrenergic receptor blockade suppresses renin release directly acting on juxtaglomerular cells [24], an impact of beta blockers on the analyzed biomarkers was not evident in our study. One explanation could be that the prescribed beta blockers were still not titrated to the optimal dosage, therefore not leading to a sufficient inhibition of renin release. However, renin secretion is a complex phenomenon and is regulated by multiple factors not limited to the sympathetic nervous system. Cluster 3, representing ACEi users, showed nearly perfect specificity, meaning that individuals who are not on ACEi would be very unlikely to be falsely classified as ACEi user. On the other hand, cluster 3 exhibited limited sensitivity, with nearly four out of ten ACEi users being missed by this classifier. Cluster 2, mainly representing ARB users, showed higher sensitivity, with seven out of ten ARB users that would be correctly identified by this classifier, but imperfect specificity, allowing some non-ARB user to enter this group.

Individuals in clusters 2 and 3 exhibited more cardiometabolic issues as compared to cluster 1. Clusters 2, encompassing ARB users, included a higher rate of individuals with DM and, consistently, individuals in this cluster aveh higher blood glucose and HbA1c levels. Individuals in cluster 3, encompassing ACEi users, had lower levels of eGFR and higher BMI. This is consistent with current clinical protocols, which prioritize assignment of ACEi and ARB for individuals with metabolic syndrome like diabetic hypertensive [25] and kidney disease [26] patients. The identified clusters were not different in terms of cortisol, potassium and sodium levels. The absence of an association with potassium, whose level can be depleted by thiazide diuretics reflects the inability of cluster analysis to discriminate those on diuretic treatment among those taking ACEi or ARB.

The penalized regression analysis of residual variability after removing the effect of the identified clusters and treatment groups, highlighted residual strong associations of sex with all three RAAS biomarkers, and age with angiotensin I and angiotensin II. Also higher eGFR, which indicates better kidney function, was associated with lower levels of all three RAAS biomarkers. A lower SBP was associated with higher angiotensin II levels, according to expectations since a drop in BP triggers RAAS to increase BP through increased release of angiotensin [27]. The detection of these associations after removal of the treatment effect and of the cluster effect, indicates the presence of additional factors acting on angiotensin I, angiotensin II and aldosterone levels that might explain the imperfect agreement between clusters obtained through unsupervised statistical analysis and objective AHD classification up on participation. In particular, there is a known differential prescription of AHD by sex [28]. However, given we adjusted the analyses for drug groups and groups were sex-matched, it is more likely that the residual association with sex is of purely biological origin.

The main feature of our study was the analysis of RAAS biomarkers typically analysed only in clinical context in a population-based scenario. This was possible thanks to a novel quantification method that allows RAAS biomarker quantification in frozen samples, thus allowing use in general population studies conducted outside of controlled clinical settings. On the other hand, limitations should be highlighted. This study relied on cross-sectional measurement which might not be reflective of the actual health status of an individual over an extended period, especially for what concerns BP. Imperfect discrimination by cluster analysis could be explained by heterogeneous counter-regulatory renin release mechanisms and by ACEi/ARB escape phenomenon, the latter dependent on individual drug response [29]. Classification based on barcode scanning of drug boxes provides great precision but our study could not take into account adherence to treatment since drug levels were not measured. This unaccounted variability may have additionally contributed to the imperfect agreement between clusters and drug groups as noncompliance is known to affect sensitivity and specificity of treatment screening [30]. While we identified a promising unsupervised procedure to identify underlying AHD treatment targeting the RAAS system, our limited sample size has prevented us an independent replication and calibration of the clustering algorithm in an independent setting is warranted. We considered enhancing the potential of the clustering by including biomarkers other than the RAAS, such as glucose, cholesterol, SBP and DBP. But the addition of these biomarkers did not improve drug identification compared to the identification achieved by using RAAS biomarkers only. Finally, our study was lacking by design the measure of urinary sodium excretion, representing dietary sodium intake.

In conclusion, our study has demonstrated that the unsupervised clustering of angiotensin-based biomarkers in previously biobanked samples is a viable technique to identify individuals on specific antihypertensive treatments from the general population, pointing to the potential application of the biomarkers as useful clinical diagnostic tools even outside of a controlled clinical setting.

## Electronic supplementary material

Below is the link to the electronic supplementary material.

Supplementary Material 1

## Data Availability

The data analysed during the current study are not publicly available due to privacy policy but are available, including the computer codes used for the analysis, from the corresponding author on reasonable request.

## Abbreviations

AHD: Anti-hypertensive drug
RAAS: Renin-Angiotensin-Aldosterone System
CHRIS: Cooperative Health Research In South Tyrol
ARB: Angiotensin type 1 receptor blockers
ACEi: Angiotensin-converting enzyme inhibitors
BP: blood pressure
DBP: diastolic blood pressure
SBP: systolic blood pressure
eGFR: estimated glomerular filtration rate
CV: coefficient of variation
PC: Principal components
ANOVA: analysis of variance
MSE: mean squared error
