# Supplementary material for: Cluster analysis of angiotensin biomarkers to identify antihypertensive drug treatment in population studies
Source: BMC Med Res Methodol. 2023 May 27;23:131. doi: 10.1186/s12874-023-01930-8 (PMC10224304; doi:10.1186/s12874-023-01930-8)
Supplement: Supplementary file 1 — Supplementary Material 1 [file 12874_2023_1930_MOESM1_ESM.pdf]

Cluster analysis of angiotensin biomarkers to identify antihypertensive drug treatment in population studies.

Supplementary material

Supplementary Figure 1: Pairwise correlations between the clinical variables tested for inclusion in the penalized regression model. 2

Supplementary Figure 2: identification of the optimal clustering solution via the Silhouette score metric. 3

Supplementary Table 1: Distributions of clinical variables across AHD groups. 4

Supplementary Table 2: Multiple pairwise-comparisons among the three clusters for clinical variables. 5

Supplementary Figure 1: Pairwise correlations between the clinical variables tested for inclusion in the penalized regression model.

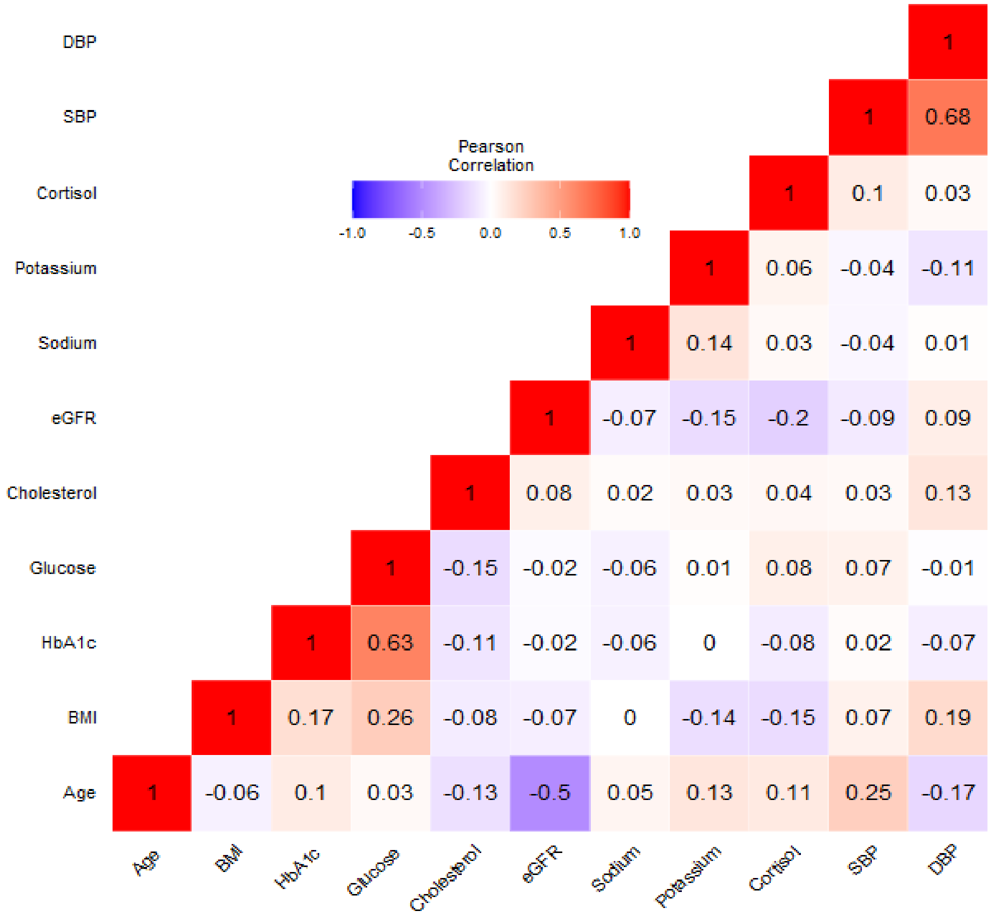

Supplementary Figure 2: identification of the optimal clustering solution via the Silhouette score metric. Extension to eight possible clusters which is equal to the number of antihypertensive treatment groups.

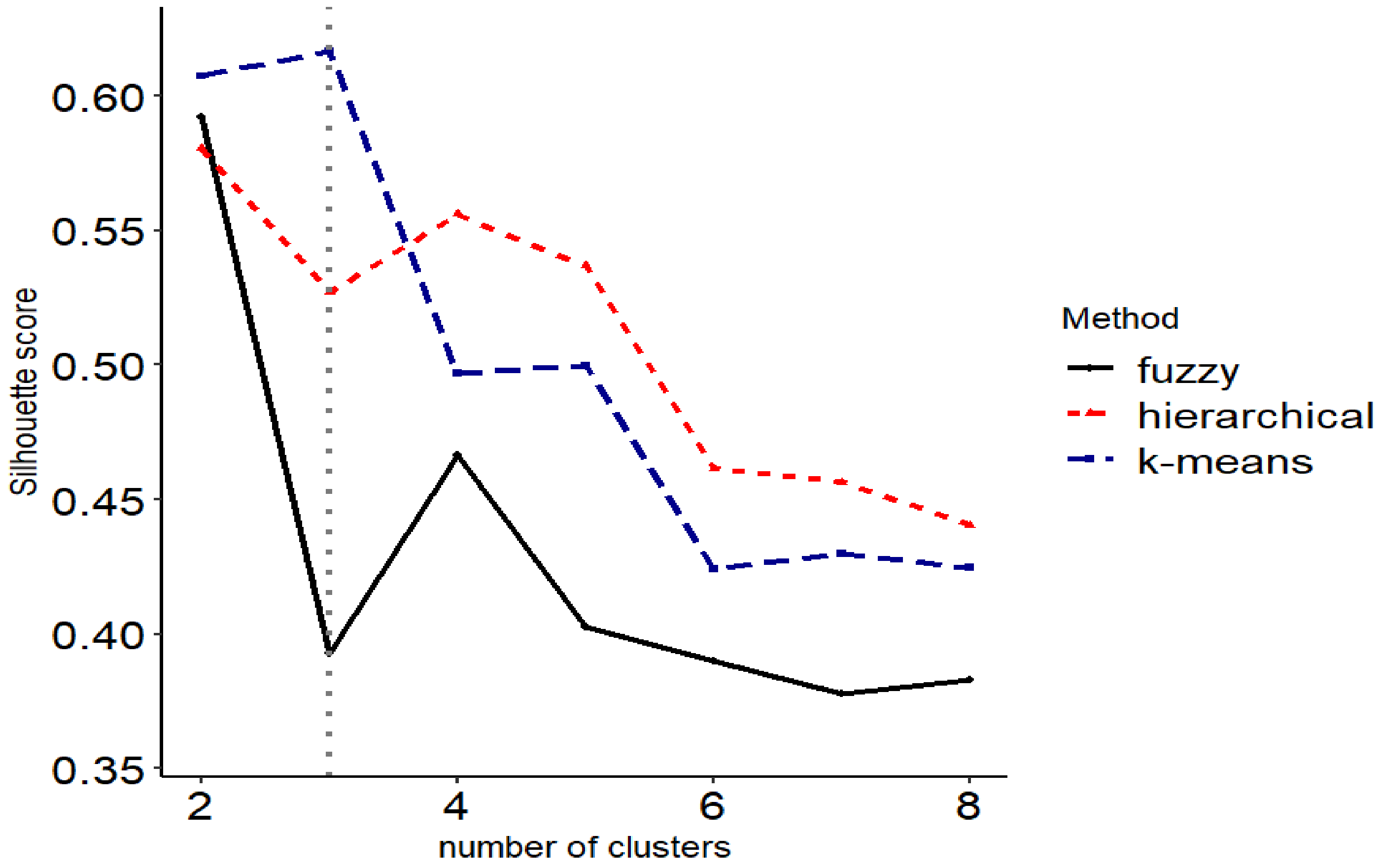

Supplementary Table 1: Distributions of clinical variables across AHD groups. Reported are means and standard deviations.

AHD group	Glucose (mg/dl)	HbA1c (%)	Cholesterol (mg/dl)	eGFR (mL/min)	Potassium (mmol/L)	Sodium (mmol/L)	Cortisol (mmol/L)
**Normotensive**	96.48(9.8)	5.86(0.37)	235.46(40.27)	78.93(11.13)	4.67(0.41)	141.19(2.41)	14.02(5.42)
**Hypertensive**	99.19(14.29)	5.87(0.41)	236.85(39.68)	80.68(12.26)	4.59(0.33)	141.12(2.09)	14.01(4.48)
**Non-AHD**	97.64(13.98)	5.85(0.46)	224.15(42.18)	77.87(13.11)	4.66(0.38)	141.25(2.29)	13.81(4.2)
**Beta blocker**	99.71(11.62)	5.93(0.38)	215.93(44)	76.8(14.25)	4.62(0.36)	140.79(2.08)	13.41(4.51)
**ACEi**	102.02(15.99)	6.08(0.58)	220.22(44.35)	74.31(15.75)	4.73(0.45)	140.78(2.32)	13.46(4.89)
**ACEi + diuretic**	105.15(23.1)	6.11(0.69)	215.26(39.4)	72.68(15.55)	4.44(0.36)	140.62(2.31)	13.75(5.09)
**ARB**	102.7(28.93)	6(0.65)	222.4(39.81)	76.21(13.8)	4.6(0.4)	140.54(1.94)	12.97(4.14)
**ARB + diuretic**	104.89(17.4)	6.08(0.56)	210.11(45.23)	74.42(13.31)	4.41(0.34)	140.95(2.79)	13.27(4.26)

Supplementary Table 2: Multiple pairwise-comparisons among the three clusters for clinical variables. The multiple pairwise-comparisons were corresponding to the variables that indicated statistically significant difference from the one-way ANOVA test of Table 3 of the main manuscript. Pairwise test 2-1 indicated test between clusters 2 (ARB enriched) and 1 (no treatment enriched), 3-1 for test between clusters 3 (ACEi enriched) and 1 and 3-2 for test between clusters 3 and 2. CI: confidence interval. The P-value was obtained from the pairwise test adjusting for multiple testing using the Tukey multiple test procedure.

Variable	Pairwise test	difference of the means between clusters	Lower 95% CI	Upper 95% CI	P-value
BMI (kg/m^2)	2-1	1.614	0.725	2.503	0.0001
	3-1	1.936	0.804	3.068	0.0002
	3-2	0.322	-0.913	1.557	0.8135
HbA1c (%)	2-1	0.13	0.03	0.231	0.0066
	3-1	0.128	0.001	0.256	0.0482
	3-2	-0.002	-0.141	0.137	0.9993
Glucose (mg/dl)	2-1	5.622	2.238	9.006	0.0003
	3-1	3.723	-0.579	8.024	0.1053
	3-2	-1.899	-6.593	2.794	0.6086
eGFR (mL/min)	2-1	-3.831	-6.434	-1.228	0.0017
	3-1	-4.791	-8.1	-1.482	0.0020
	3-2	-0.96	-4.569	2.649	0.8067
SBP (mmHg)	2-1	-5.582	-8.866	-2.298	0.0002
	3-1	-0.449	-4.624	3.725	0.9654
	3-2	5.133	0.578	9.688	0.0226
DBP (mmHg)	2-1	-1.713	-3.446	0.02	0.0435
	3-1	-1.316	-3.519	0.887	0.3399
	3-2	0.397	-2.007	2.8	0.9205
